# Enhancing Automatic Classification of Hepatocellular Carcinoma Images through Image Masking, Tissue Changes, and Trabecular Features

**DOI:** 10.1155/2014/726782

**Published:** 2014-12-17

**Authors:** Maulana Abdul Aziz, Hiroshi Kanazawa, Yuri Murakami, Fumikazu Kimura, Masahiro Yamaguchi, Tomoharu Kiyuna, Yoshiko Yamashita, Akira Saito, Masahiro Ishikawa, Naoki Kobayashi, Tokiya Abe, Akinori Hashiguchi, Michiie Sakamoto

**Affiliations:** ^1^Department of Information Processing, Interdisciplinary Graduate School of Science and Engineering, Tokyo Institute of Technology, 4259-S1-17 Nagatsuta, Midori, Yokohama, Kanagawa 226-8503, Japan; ^2^Global Scientific Information and Computing Center, Tokyo Institute of Technology, 2-12-1-I7-6 Ookayama, Meguro-ku, Tokyo 152-8550, Japan; ^3^Medical Solutions Division, NEC Corporation, 5-7-1 Shiba, Minato-ku, Tokyo 108-8001, Japan; ^4^Faculty of Health and Medical Care, Saitama Medical University, 1397-1 Yamane, Hidaka-shi, Saitama 350-1241, Japan; ^5^Department of Pathology, School of Medicine, Keio University, 35 Shinanomachi, Shinjuku-ku, Tokyo 160-8582, Japan

## Background

Hepatocellular carcinoma (HCC) is a malignant tumor with hepatocellular differentiation and one of the most common cancers in the world. This type of cancer is often diagnosed when the survival time is measured in months causing high death rates [1]. For the purpose of supporting histopathology diagnosis of HCC, we have developed an experimental system of “feature measurement software for liver biopsy” [2]. The system provides pathologists with the quantitative measurement of tissue morphology using a digital slide of hematoxylin-eosin (HE) stained liver tissue specimen, as well as the HCC detection based on those measurement results. In this study, we are focusing on the classification process of HCC images in the system. Previously, Kiyuna et al. [3] had introduced an automatic classification of HCC images based on 13 types of nuclear and structural features, where each feature consists of 6 statistical distributions. In order to improve the classification performance, we have developed methods to segment the liver tissue and quantify additional tissue features such as trabecular morphology [4]. This paper reports the evaluation results on the impact of the segmentation and the additional features in the HCC detection performance.

## Method

We enhanced the classification process presented in [3] by including 11 features of tissue changes (i.e., features related to fatty change, cytoplasm colors, cell clearness index, and stroma) and 10 features of trabecular (e.g., nuclei-cytoplasmic ratio, irregularity of sinusoid, and trabecular arrangements). Furthermore, we apply a mask obtained by the stroma segmentation before calculating the 13 types of nuclear and structural features such that those features are derived from hepatocytes only, thus generating in total 177 features. The experiments were performed on a collection of region-of-interest (ROI) images extracted from HE stained whole slide images (WSI), consisting of 1054 ROIs of HCC biopsy samples (504 negatives and 550 positives) and 1076 ROIs of HCC surgically resected samples (533 negatives and 543 positives). In the process, we made some combinations on the sets of features and sets of training data from both biopsy and surgery samples. As for the classification, we used 5-fold cross validation support vector machine (SVM) with LibSVM as our library.

## Results

The results of classification experiment are summarized in Table 1. Our experiments show that combinations of the new features with the nuclei and structural features can improve the accuracy for about 1–3% depending on the type of training and test data. For example, in biopsy samples, the sensitivity is improved from 84.7% to 88.2% while the specificity is unchanged (91.9%). Furthermore, in surgery samples, the detection rate for the well-differentiated tumors (Edmondson grade 1) is improved from 65.0% to 77.5% by the addition of new features. Nevertheless, the masking process on the nuclei features brings different effect on biopsy and surgery samples, but it facilitates the reliability of the nuclei features since falsely detected nuclei are removed from the quantification.

## Conclusion

The combination of nuclear, trabecular, and other tissue features enables improved classification rate in HCC detection using SVM. Even though the image characteristics are different in biopsy and surgically resected samples, the same classification system gives good performance in both samples. The HCC classification scheme introduced in this paper is implemented in the prototype “feature measurement software for liver biopsy,” and the probability of HCC is visualized for every ROI in the WSI. It will support pathologists in the HCC diagnosis along with the quantitative measurements of tissue morphology.

## Figures and Tables

**Table 1:** Experiment results.

Training data	Test data	Set of features	Sensitivity	Specificity
Biopsy sample	Biopsy sample	78 nuclei (unmasked)	86.36%	88.29%
Surgery sample	Surgery sample	78 nuclei (unmasked)	88.21%	87.99%
Combination of biopsy and surgery sample	Biopsy sample	78 nuclei (unmasked)	84.73%	91.87%
		78 nuclei (masked)	85.27%	90.67%
		72 nuclei (masked) + 21 new features	88.18%	91.87%
	Surgery sample	78 nuclei (unmasked)	88.95%	87.62%
		78 nuclei (masked)	89.50%	87.62%
		72 nuclei (masked) + 21 new features	91.34%	89.68%
